# A Comparative Analysis of Signal Decomposition Techniques for Structural Health Monitoring on an Experimental Benchmark

**DOI:** 10.3390/s21051825

**Published:** 2021-03-05

**Authors:** Marco Civera, Cecilia Surace

**Affiliations:** 1 Department of Mechanical and Aerospace Engineering—DIMEAS, Politecnico di Torino, Corso Duca degli Abruzzi 24, 10129 Turin, Italy; 2 Department of Structural, Geotechnical and Building Engineering—DISEG, Politecnico di Torino, Corso Duca degli Abruzzi 24, 10129 Turin, Italy; cecilia.surace@polito.it

**Keywords:** signal processing, structural health monitoring, adaptive mode decomposition methods, time-frequency analysis, damage detection, empirical mode decomposition, Hilbert Vibration Decomposition, variational mode decomposition

## Abstract

Signal Processing is, arguably, the fundamental enabling technology for vibration-based Structural Health Monitoring (SHM), which includes damage detection and more advanced tasks. However, the investigation of real-life vibration measurements is quite compelling. For a better understanding of its dynamic behaviour, a multi-degree-of-freedom system should be efficiently decomposed into its independent components. However, the target structure may be affected by (damage-related or not) nonlinearities, which appear as noise-like distortions in its vibrational response. This response can be nonstationary as well and thus requires a time-frequency analysis. Adaptive mode decomposition methods are the most apt strategy under these circumstances. Here, a shortlist of three well-established algorithms has been selected for an in-depth analysis. These signal decomposition approaches—namely, the Empirical Mode Decomposition (EMD), the Hilbert Vibration Decomposition (HVD), and the Variational Mode Decomposition (VMD)—are deemed to be the most representative ones because of their extensive use and favourable reception from the research community. The main aspects and properties of these data-adaptive methods, as well as their advantages, limitations, and drawbacks, are discussed and compared. Then, the potentialities of the three algorithms are assessed firstly on a numerical case study and then on a well-known experimental benchmark, including nonlinear cases and nonstationary signals.

## 1. Introduction

Real-life signals from vibrating structures are difficult to investigate, mainly due to the presence of measurement noise, their nonstationarity, and the noise-like distortions induced by the presence of structural nonlinearities [1]. Nevertheless, the dynamic response of a target system can disclose its mechanical properties—that is to say, its stiffness and/or mass [2]. This is also known as (linear or nonlinear) System Identification (SI/NLSI). For unchanged operational, environmental, and boundary conditions (i.e., in absence of confounding influences), the variations of these properties can be directly related to the development of damage [2]. Therefore, with a proper comparison between the current situation and the known “normal” baseline model of the system, vibration-based damage detection and Structural Health Monitoring (SHM) can be performed.

In this sense, the purpose of any decomposition technique is to isolate and extrapolate a Defect Signal Mode (DSM) from the wide-band recordings of the structural response. Then these DSMs can be used on their own or (most commonly) further post-processed individually to extract one or more Damage-Sensitive Features (DSFs) from each mode. Then, surrogate models can be defined from the DSMs and/or the DSFs collected during the structure normal conditions (the best practice would be to include data recorded during varying environmental and operational conditions to make a robust model, less subject to false alarms). This is generally performed through pattern recognition and Artificial Intelligence (AI) approaches (techniques based on Bayesian Machine Learning are particularly well-suited [1]). Finally, basic outlier detection statistical tools can be applied to discover damage-related anomalies.

The basic idea behind signal decomposition is to “break down” a complicated signal into simpler, yet similar, components, possibly rapidly and efficiently. These components—or modes—should be as independent from each other as possible, not too differently from the eigenmodes obtainable from the modal analysis of a time-independent, linear system. Eventually, they should preferably have some sparsity properties—e.g., being bandwidth limited in the frequency domain and compact around a centre frequency. A requisite is that the combination of all these components can return the original signal ideally without losses of the carried information (i.e., with minimum least-square error). All these requisites make these modes similar, but not necessarily identical, to the linear normal modes (LNMs), and the concepts should not be confused [3].

Then, these “modes” can be exploited in several manners. For instance, very low- or very high-frequency content, unrelated to the actual mechanical properties of the investigated structure, can be discarded. These tasks are referred to as denoising and baseline drift removal; both procedures are widely popular for SHM, even if one should use particular care since the removal of nonstationary components can lead to missing data information, especially for civil engineering applications when the input excitation is a ground motion [4]. Other uninteresting modes, irresponsive to damage and/or governed by other external factors (such as temperature, seasonal changes, and vibrations induced by operating machinery, pedestrians, or vehicles) can be removed as well. Nonstationary components can also be dismissed, trying to highlight time-independent mechanisms or features [5]. Finally, some modes can be found to be more sensitive to damage than others; thus, it is easier to detect small changes (e.g., due to crack development in its earlier stages) in a single component rather than in the complete signal, as these small variations may be masked by the other damage-insensitive information. 

### Adaptive Mode Decomposition Methods

Several algorithms have been proposed in recent years for the purposes highlighted above. A quite complete and up-to-date review can be found in Ref. [6] specifically for fault diagnosis and condition monitoring. However, at the current state, no methodical analysis has been performed on these approaches from an SHM point of view. Many review papers only enlist the applications found in the literature, reporting the findings and statements of other researchers. Other review papers perform some limited testing, generally applying the algorithms on one or few (often synthetic) signals, and with minimal commentary. The purpose of this direct comparison is to address the accuracy and efficiency of such methods for the extraction of DSFs and the subsequent Machine Learning training. Among the many proposals found in the scientific literature, the most well-known approaches include the Empirical Mode Decomposition (EMD, [7]), the Local Mean Decomposition (LMD, [8]), the Local Characteristic scale Decomposition (LCD, [9]), the Hilbert Vibration Decomposition (HVD, [10]), the Empirical Wavelet Transform (EWT, [11]), and the Variational Mode Decomposition (VMD, [12]).

All of them are more or less empirically based or retain some theoretical principles that justify the way they operate. Their common goal is to allow data-adaptive time-frequency analysis for nonstationary time histories (THs) of linear or nonlinear systems.

Of the six approaches mentioned above, the LCD follows the same framework as the EMD, with the only relevant difference lying in the instantaneous mean extraction [6]. However, this step as well uses an approach similar to the EMD, based on cubic spline interpolation, with all the same technical issues.

Similarly, the LMD as well suffers the same shortcomings as the EMD (which will be discussed later). Moreover, it has been proved to be significantly affected by the choice of the smoothing and step size parameters [6]. For all these reasons, LCD and LMD have not been considered in this comparative analysis. On the other hand, the EWT algorithm has been tested on the same numerical and experimental datasets presented in this paper. However, the specific findings are deferred to a future similar work, focused specifically on wavelet-based (adaptive or not) techniques. Due to the authors’ intent to analyse and compare the techniques in depth, some other interesting approaches e.g., the Intrinsic Time-scale Decomposition (ITD, [13]), had to be omitted because of space limitations; these will be addressed in future works as well.

Hence, the authors have selected a shortlist of three algorithms, which have reached undisputable popularity in many engineering fields and differ substantially from each other. These are the EMD, the HVD, and the VMD. All of them have been successfully applied for fault and damage detection (see e.g., References [14,15]). These three methods are here quantitatively compared. Firstly, a dataset of numerically simulated vibration is utilised to estimate the robustness to artificially added measurement noise. Then, a well-known, well-documented case study—a three-storey frame model developed at the Los Alamos National Laboratory [16]—is used as a benchmark. This simple yet compelling case study includes nonlinear distortions, stiffness reductions, and added masses. Please note that the algorithms reviewed here are neither limited to time series nor one-dimensional or single-channel data analysis; however, only this case will be addressed here, even when the respective bivariate or multivariate extensions exist (e.g., [17,18]).

The rest of this paper is organised as follows. The theory and mathematical formulation behind the three alternatives investigated here are reported in Section 2. The advantages and limitations of each approach are recalled as well. Section 3 describes the experimental benchmark utilised for this comparative study, as well as the parameters selected for this aim. Section 4 discusses the results. This work ends with the conclusions.

## 2. Theoretical Background

In this section, the theory behind the three decomposing techniques of interest is addressed. As recalled, all these approaches break down the target signal into various components. This can be performed directly in the time domain (such as for the EMD and derived techniques), in the frequency domain (e.g., the VMD), or in the time-frequency (or time-scale) plane. The rationale behind frequency- and time-frequency-based decomposition techniques is that most real-world signals have compact, band-limited Fourier spectra; methods of this kind implicitly assume that the independent components of the signal have narrow-band properties and (generally) well-distinct spectrum supports [19]. What differentiates the approaches here reviewed from the classic Fourier transform (FT) is that they all allow the investigation of time-varying (nonstationary) signals. The techniques are analytically or empirically defined, i.e., they operate according to an algorithm but lack a proper mathematical definition. That provides them with some degrees of data adaptability.

The three methodologies are described in detail in the following subsections. The discussion starts with the EMD (and subsequent variants), the HVD, and the VMD, in this order. Since the estimation of the instantaneous frequencies (IFs) and amplitudes (IAs) is necessary for the analysis of the resulting modes, the definition of the Hilbert transform (HT) is recalled in the subsection dedicated to the EMD.

### 2.1. Empirical Mode Decomposition (EMD) and Derived Algorithms

Since its first introduction in 1998 [7], the Empirical Mode Decomposition (EMD) has been widely studied and applied in many fields. For structural purposes, it has been exploited for System Identification (e.g., [20,21]), also by approximating the eigenmodes of bridges for Operational (i.e., output-only) Modal Analysis [22], even during extreme events [23]. It has been applied for the SHM of steel frame structures [24] as well, among many other similar applications. The technique is particularly appreciated for the condition monitoring of rotating machinery (see Ref. [25] for a dedicated overview). Some other useful reviews for SHM purposes can be found in [26], in the very recent Ref. [14], and in Chapter 14 and 15 of Ref. [27].

The EMD is used to decompose the target signal into a finite (and generally small) number of natural oscillatory modes, which are called IMFs (Intrinsic Mode Functions) [7]. The IMFs are oscillations incorporated in the original signal, and their amplitude and frequency are not constant over time, unlike the harmonics that are obtained with the Fourier transform [28]. That is to say, the IMFs preserve their instantaneous frequency information. 

With the Hilbert Spectral Analysis (HSA), the EMD forms the basis of the Hilbert–Huang Transform (HHT) for the estimation of the IFs and of the time-frequency distribution of the signal amplitude (i.e., the Hilbert–Huang Spectrum $H\left(\omega ,t\right)$). If the HSA was performed directly on the whole (non-decomposed) signal, it could return physically impossible results such as negative frequencies [28]. Instead, the use of IMFs enforces physically meaningful results. This is achieved by imposing some rules on how an IMF is defined. Specifically, for a function to be considered an Intrinsic Mode Function, it must satisfy the following two conditions:

1. The number of local maxima and minima and the number of points where it assumes a zero value must be equal or at most differ by one unit;
2. The local mean, defined as the mean of the upper hand lower envelopes, must be zero.

**Figure 1:**
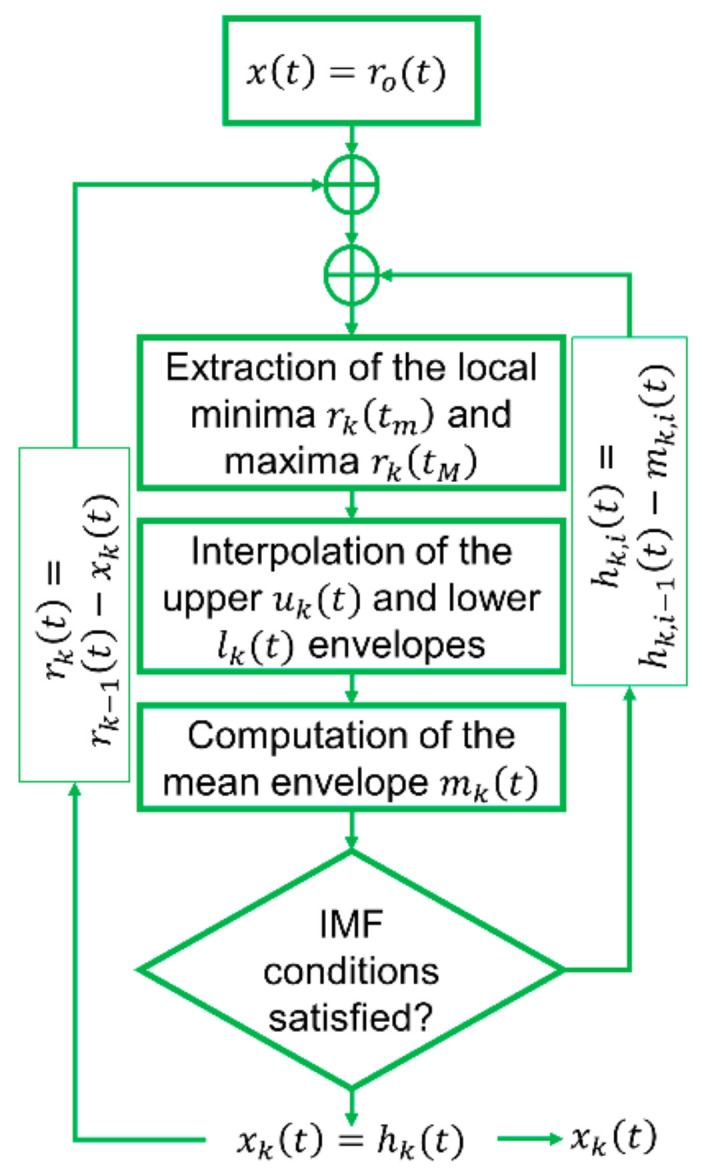
Flowchart of the Empirical Mode Decomposition (EMD) algorithm.

Thus, the IMFs are essentially zero-mean AM-FM components. Then, the original signal can be reconstructed directly as the sum of all IMFs in the time-domain [7]. Importantly, the IMFs have no analytical formulation [29]. This is related to the EMD being an algorithm, i.e., a set of arbitrary (and empirical, as the name suggests) rules that can be applied to a dataset and lack a well-defined theoretical basis (differently from, e.g., the FT). The EMD decomposition procedure, also known as the “sifting process” [30], is described in details in Ref. [7]. The complete description is omitted here for brevity; a graphical depiction is portrayed in Figure 1, where, at any k-th step, the sifted signal (i.e., the residual) is called $r_{k}\left(t\right)$ (for k=0, $r_{k=0}\left(t\right)=x\left(t\right)$). The average between the upper and lower envelopes returns $m_{k}\left(t\right)$, and the difference $r_{k}\left(t\right)-m\left(t\right)$ is iterated over the additional counter i until it satisfies the two conditions by which an IMF is defined. At that point, $x_{k}\left(t\right)$ is the k-th IMF and the new residual is calculated. The algorithm stops when the K-th residual $r_{K}\left(t\right)$ is a monotone function or a function with less than two local minimum or maximum points.

A major technical limitation of the basic EMD definition is the issue of the so-called “mode-mixing” when the method is applied to signals affected by measurement noise (or noise-like nonlinear disturbances). This phenomenon involves the over-decomposition of a signal, and it leads the algorithm to extract more IMFs than the number of oscillatory modes that make up the original signal. The mode-mixing is due to EMD being a completely data-driven technique: the addition of noise involves the creation of “fictitious” maximum and minimum points that are identified by the algorithm and interpolated. This causes even a mono-component signal such as a simple sinusoid to be broken down into several sub-signals when affected by noise [31]. This high sensitivity to noise limits the reliability of an EMD-based signal monitoring scheme. However, to alleviate the mode mixing problem, a series of improvements have been introduced, representing the direct evolution of the EMD algorithm:

1. The Ensemble Empirical Mode Decomposition (EEMD; [31]);
2. The Complete Ensemble Empirical Mode Decomposition with Adaptive Noise (CEEMDAN; [32]).

These two improvements to the basic EMD can be found in the respective references. Here, in this study, the CEEMDAN algorithm was applied to the benchmark dataset of interest. This involves the use of $N_{R}$ realisations of White Gaussian Noise (WGN) defined by the noise standard deviation ϵ. This improved variant is supposed to strongly reduce the detrimental effects of noise, even if at the cost of being more computationally intensive.

#### The Hilbert Transform

Once all the IMFs have been defined, the Hilbert transform [7] can be applied to them as:

(1) $$y_{k}\left(t\right)=\frac{1}{\pi }p.v.\int_{-\infty }^{\infty }\frac{x_{k}\left(\tau \right)}{t-\tau }d\tau$$

where p.v. denotes the Cauchy principal value. Equation (1) can be considered as the convolution of the *k*-th mode with 1/t, which is intended to emphasise the instantaneous properties of $x_{k}\left(t\right)$. To define their time-varying IF and envelope of the signal, it is then sufficient to compute

(2) $$\omega_{k}\left(t\right)=\frac{d\varphi \left(t\right)}{dt}$$

where $\varphi \left(t\right)=\arctan\frac{y\left(t\right)}{x\left(t\right)}$ is the instantaneous phase, and

(3) $$A_{k}\left(t\right)=\sqrt[{2}]{x_{k}^{2}\left(t\right)+y_{k}^{2}\left(t\right)}.$$

is the IA. For all the other details concerning the HT, the interested audience is referred to the extensive tutorial review presented in Ref. [33].

Please note that several other techniques are available for the estimation of instantaneous parameters. Indeed, the HT has some well-known flaws—it is limited by the Bedrosian theory, can return negative instantaneous frequencies, and suffers from energy leakage at the signal edges, making the estimation of the IF and IA unreliable close to them [34]. The HT is also very sensitive to noisy data since it requires the derivative of both the real and the imaginary part of the transformation [35]. The same issues affect similar procedures such as the Wigner–Ville Distribution (see Ref. [36] for a more detailed discussion). A much better estimation of the IF can be achieved e.g., by simply considering the time sequence of the signal zero-crossing points [35,37].

The potential alternatives include the Energy Separation (ES, [38]), the Teager Energy Operator (TEO) [39], the Normalised Hilbert Transform (NHT), the Generalised Zero Crossing (GZC), and the Direct Quadrature (DQ) algorithms (the last three are all presented in Ref. [34]), plus other options derived from them, e.g., the Quadrature Derivative based Normalised HT (QDNHT, [40]). These approaches, among many others, have been proposed to address some of the HT shortcomings. However, this point does not fall into the specific aim of this study and it will not be further discussed for brevity; a detailed comparison can be found e.g., in Ref. [6].

### 2.2. Hilbert Vibration Decomposition (HVD)

The Hilbert Vibration Decomposition (HVD) was introduced by Feldman in 2006 [10] for the extraction of components (modes) from nonstationary broadband signals. The method is similar to EMD (and derived techniques) in the sense that it is still a recursive decomposition scheme performed iteratively on the residual signal from the previous step [12]. The HVD approach relies on the Hilbert transform (HT), as the name suggests. The interested reader is referred to Feldman’s book [41] for a deeper discussion. At any iteration, the dominant (i.e., most energetic) vibration is extracted; its IF is estimated as the average frequency obtained from the HT. This is intended to enforce that any inherent component has mathematical and physical significance, rather than merely satisfying some user-defined requisites [10].

The HVD method has been proposed for SHM applications [42], even if its main field of application remains the SI of nonlinear mechanical systems [43]. The HVD algorithm has been validated on nonlinear systems with single or multiple degrees of freedom (e.g., in Ref. [44] for a two-DoF system), even if not yet on large real-life structures [45].

The basic assumption is that the original signal is formed by a superposition of harmonic or quasi-harmonic functions. If the signal is multi-component, then it can be decomposed into several, distinct mono components [46]; for continuous time, this operation can be intended as

(4) $$f\left(t\right)=\overset{K}{\underset{k=1}{\sum }}x_{k}\left(t\right)=\overset{K}{\underset{k=1}{\sum }}A_{k}\left(t\right)\cos\left(\int \omega_{k}\left(t\right)dt\right)$$

where $f\left(t\right)$ is the whole signal and $x_{k}$ are its single-mode terms defined by their instantaneous amplitudes and frequencies ($A_{k}\left(t\right)$ and $\omega_{k}\left(t\right)$, respectively). Then, the instantaneous phase of the signal is given by $\varphi_{k}\left(t\right)=\int \omega_{k}\left(t\right)dt$. Importantly, the authors of the EMD algorithm did not provide a similar analytical definition for their IMFs. Every $x_{k}\left(t\right)$ is assumed to be a simple waveform, with slowly varying $A_{k}\left(t\right)$ and $\omega_{k}\left(t\right)$. This assumption has important repercussions on the properties of the extracted modes; these will be better addressed later. The HVD iterative algorithm can be summarised in three sequential steps. Specifically:

1. The instantaneous frequency of the vibration component with the largest energy content (i.e., amplitude) is estimated;
2. The envelope of the same is extracted;
3. The component is subtracted from the signal.

**Figure 2:**
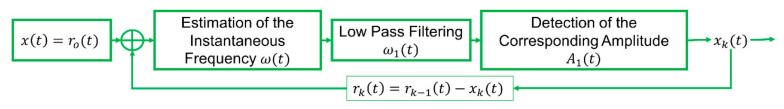
Flowchart of the Hilbert Vibration Decomposition (HVD) algorithm.

Then, the residual is passed to the next iteration until the desired number *K* of modes is extracted. A block diagram description of the algorithm is reported in Figure 2. One can see that a major conceptual difference between EMD and HVD is that the former algorithm independently estimates IF (via zero-crossing) and IA, while the latter only estimates the IF and then extracts the corresponding IA. This choice has both advantages (such as a better frequency resolution) and disadvantages [47].

**Figure 3:**
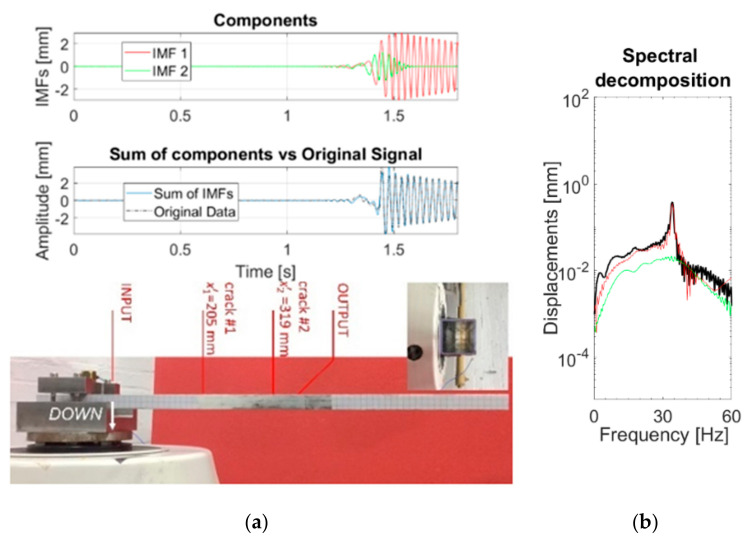
Results from the target signal (displacement time history). (**a**) IMFs (Intrinsic Mode Functions) in the time domain and experimental setup; (**b**) IMFs in the frequency domain.

A more complete and detailed description of the whole algorithm can be found in the original work of Feldman [10]. Figure 3 reports an example of an application of the HVD technique to an experimental dataset. K=2 was imposed. The signal, a displacement TH, comes from a cantilevered aluminium box beam, with two saw-cuts intended to mimic surface open cracks and subject to 11-μs-long triangular impulses. The full description of the setup can be found in Ref. [48]. In this example, the first IMF can be linked to the steady-state free oscillation of the beam, while the second one captures the transient effects of the input, similarly to what expected for the forced nonstationary response of the inspected system [10]. However, far from the input, the transient term fades away. By increasing K, no other relevant IMF arises, hinting at a single-component linear behaviour (as expected for this specific structure, which behaved linearly even after damage insertion).

### 2.3. Variational Mode Decomposition (VMD)

Even if not yet as widespread as EMD, the VMD algorithm (proposed in 2014 by Dragomiretskiy and Zosso [12]) has been already extensively applied in Structural Engineering. Ref. [49] applied it for the SI of time-dependent systems, such as a bridge with a passing vehicle (simulated on a laboratory experiment). Ref. [50] validated it for the output-only approximation of the vibrational modes of a pedestrian bridge, proposing the approach for SHM uses as well. Ref. [51] applied the VMD for the fault detection on rotating machinery and for NLSI, showing how VMD returned separate modes corresponding to the frequency of the driving force, the first superharmonic (2×), and the most pronounced subharmonic (1/3×), plus the higher-frequency content.

The VMD assumes the definition of “modes” introduced by the HVD [10] i.e., as seen before, amplitude-modulated–frequency-modulated (AM–FM) sub-parts of the whole signal, which can be reconstructed by simply summing them up in the time domain. By recalling Equation (4), these modes must satisfy the following conditions: (1) the envelope must be non-negative ($A_{k}\left(t\right)\geq 0\;\forall t$); (2) the phase must be non-decreasing—i.e., it must have a non-negative first-time derivative ($\varphi \prime_{k}\left(t\right)\geq 0\;\forall t$); (3) both the instantaneous amplitude $A_{k}$ and the instantaneous frequency $\omega_{k}$ must vary much slower than the instantaneous phase $\varphi_{k}$. This concept is shared not only by the HVD but also by e.g., the synchrosqueezed wavelet transform and by the EWT, among many other similar approaches. The rationale for this additional requirement is that for an interval $\left[t,t+∆t\right]$ long enough (i.e., with ∆t at least circa equal to $2\cdot \left(\frac{2\pi }{\omega_{k}}\right)$), the *k*-th IMF can be approximated to a pure harmonic function with time-varying (instantaneous) amplitude $A_{k}\left(t\right)$ and frequency $\omega_{k}=\varphi \prime_{k}\left(t\right)$. This is quite different from the classic EMD definition of IMF, where the modes’ supports may span over a large range of frequencies with more peaks. Since all the original IMF requirements are still met, this alternative definition can be seen as a restriction of EMD’s IMFs [12]. The outcome is a limited (in practical terms) bandwidth IMF, which is compact around a centre frequency. Please note that the spectral support of an FM signal is theoretically infinite; yet, the core of the signal’s power content may be bounded according to Carson’s rule [52], which can be here used to define the total IMF bandwidth (with a correction accounting for the frequency of the amplitude modulation) [12]. For this reason, the modes defined as in Equation (4) are also known as Band-Limited Intrinsic Mode Functions (BLIMFs). The signal (and therefore its constituent modes) is also required to be integrable and square-integrable up to second derivatives [12]. Then, the IFs of time-dependent modes can be estimated employing the HT on the extracted BLIMFs, not differently from the EMD and derived approaches.

The basic scheme of the VMD algorithm is recalled here; a more detailed description of the complete algorithm can be found in the original work [12]. In brief:

1. For each mode $x_{k}\left(t\right)$, the associated analytic signal is computed employing the Hilbert transform. The result is a unilateral frequency spectrum (the negative frequencies of the FT spectrum are discarded, as they do not have physical meaning and are superfluous due to the Hermitian symmetry);
2. Each mode’s spectrum is re-centred on the estimated centre (angular) frequency $\omega_{k}$ by mixing with a tuned exponential function;
3. The bandwidth is eventually estimated through the $H^{1}$ Gaussian smoothness of the demodulated signal, which is the squared Euclidean norm ($L^{2}$-norm) of the gradient.

These three steps translate into a constrained variational problem, the complete analytical explanation of which can be found in the original work of Dragomiretskiy and Zosso [12]. Importantly, the number K of modes to be extracted is chosen a priori by the user (as for the HVD).

### 2.4. Qualitative Comparison of the Techniques

The main characteristics of the three algorithms are summarised in Table 1. The EMD, being empirical by definition, operates within an “algorithmic ad-hoc nature” [12] and lacks any physical justification and a a proper mathematical definition (both of the algorithm and the extracted IMFs). The other two techniques are more defined from an analytical point of view. VMD and HVD share the same requisites for band-limited IMFs, which are slightly different (and stricter) from the original IMF definition adopted for the EMD. The additional requirement of slowly time-changing properties limits the applicability of the methods; however, they still cover many real-life scenarios. On the other hand, the BLIMF definition better enforces quasi-orthogonal signal decomposition [51].

The EMD/EEMD/CEEMDAN algorithms return a fixed number of IMFs (based on the time-varying properties of the time series). The HVD and VMD require the user to pre-define the number *K* of modes to be extracted. Both options have pros and cons. Essentially, the more a decomposing technique is data-driven, the more likely it may end up being overfitted over a single signal, making the comparison between different signals more problematic. Conversely, without some specific knowledge, it may be difficult to estimate a priori the number of independent modes.

Ref. [53] compared the VMD and EMD for the analysis of seismic signals, claiming that the former outperforms the latter. For the same application, Ref. [54] suggested using EMD and HVD combined, as the former is more reliable for high-frequency content and the latter is more reliable for high energy, low-frequency ones. Ref. [55] highlighted a larger accuracy of support-vector machines (SVM) classifiers trained on VMD-extracted BLIMFs in comparison to the ones trained with other signal components. Therefore, the VMD is expected to compare well with respect to the other options (this point was confirmed by the findings of this research, as it will be shown later).

For a deeper analysis, the full list of advantages and disadvantages of the three methods, as mentioned in the reviewed papers, is reported in the three tables of Appendix A.

## 3. The Numerical and Experimental Case Studies

For the sake of this comparative analysis, two datasets, one numerical and one experimental, have been used. The former case study was investigated to properly address the performances of the three techniques on ideal conditions, i.e., with noise-free vibrations. Then, the effects of artificially added WGN and sensor placement have been studied as well. All the other investigations were performed directly on the experimental data.

### 3.1. The Numerical Dataset

The first dataset is made up of the simulated response of the Finite Element Model (FEM) of the spar for the prototype XB-1 wing [56]. Its geometry and mechanical properties are reported in Table 2. Due to the high flexibility of the structure, minimally invasive and non-contact measurements were needed to investigate the structure; specifically, the video processing procedure of Ref. [57] was applied for the FE Model Updating.

**Figure 4:**
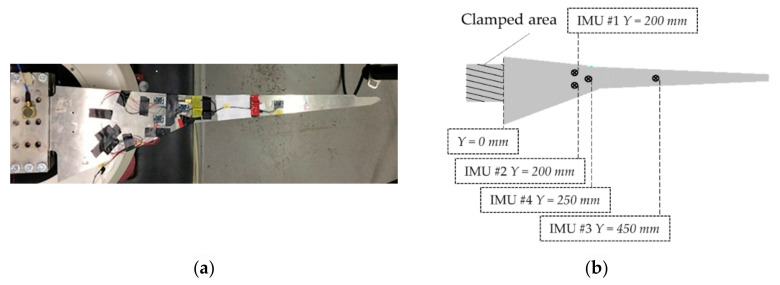
(**a**) The original experimental setup; (**b**) scheme of the wing spar FEM (top view). Y indicates the position on the main axis of the beamlike structure.

The linear FE analysis was run on ANSYS^®^ Mechanical APDL™ emulating the experimental setup described in Ref. [58]—i.e., clamped-free, with Inertial Measurement Units (IMUs) attached to the four points marked in Figure 4.

A random noise excitation was applied to the wingtip, perpendicularly to the spar with a peak acceleration of 1.5 g. A total of 1280 data points per output channel and simulation were collected, for a sampling frequency of 256 Hz and a duration of 5 s. The first flapwise bending mode was clearly identifiable from the noise-free signals at 5.49 Hz. To a lesser extent, the second mode at 23.16 Hz can be noticed as well. The response signals were then artificially corrupted with increasing levels of additive WGN, which were defined from 0 to 0.01 standard deviations of the original signal in steps of $10^{-4}\;\sigma$. The MatLab randn() function was utilised for this purpose.

### 3.2. The LANL Benchmark

**Figure 5:**
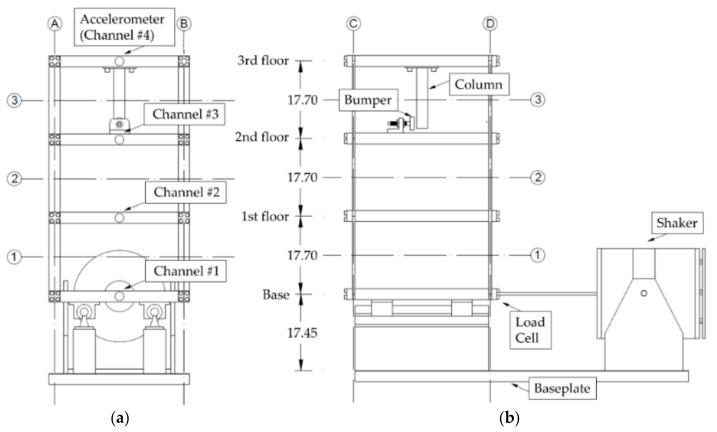
The experimental test setup schematics. (**a**) Front view; (**b**) side view.

The comparison of the signal decomposition techniques has been mainly performed on a well-known experimental benchmark, which was proposed by the Los Alamos National Laboratory (LANL) and fully described in References [16,59]. The main points are here recalled for completeness. The test structure is a three-storey shear-type frame, excited horizontally at the moving base. A scheme of the test frame, adapted by the authors from Ref. [59], is reported in Figure 5 (the shaker and the sensors’ positions are highlighted). Please note that only one output channel per floor was available. However, this is sufficient to fully characterise the model under the assumption of shear-type frame behaviour, which is well justified here. The model presents two typical forms of detectable damage-induced features: (1) a localised reduction of the stiffness, and (2) a pointwise source of nonlinearity. It also introduces added masses to simulate damage-unrelated operational changes [59].

The clapping behaviour, intended to mimic a so-called “breathing crack”, was achieved by introducing a bumper–column bilinear mechanism. The nonlinearity of the system is governed by moving the bumper closer to the column’s tip, such that it comes in touch with the bumper at lower input amplitudes. It is important to state that in a real-life structure with breathing surface cracks, the stiffness will be equal to the one of the pristine structure on the closing direction and reduced on the opening side (see Ref. [60] for a more detailed discussion). In this setup, the stiffness is unchanged in the opening direction and augmented in the opening one. This returns a global increase of the total stiffness for the “damaged” scenarios, rather than a decrease, as one would expect. Nevertheless, this setup (1) introduces damage-induced-like nonlinear effects in the vibrational response and (2) generates frequency shifts that are detectable with outlier detection approaches.

#### The Experimental Campaign

The total duration of each acquisition is 25.60 s, with a sampling frequency $f_{s}=320$ Hz. Thus, the response signals result in an 8192-points time series; their frequency spectra consist of 3600 frequency samples, up to a maximum frequency of 140.6 Hz at a resolution of 0.0391 Hz. These sampling parameters were deemed adequate for the intended uses (being the highest natural frequency about half of the available bandwidth upper bound) [59].

The input force (a band-limited random excitation, ranging from 20 to 150 Hz) is reported in newtons, while the four output channels captured the response in terms of acceleration time histories (here reported in g, with $1\;g=\;9.81\;m/s^{2}$). The campaign included a baseline scenario and sixteen alterations (all reported in Table 3).

Fifty realisations per scenario were performed. For the sake of this study, the experiments are considered as follows: Case 1 is the pristine structure that serves as a baseline model; Cases 2 and 3 present damage-unrelated frequency shifts due to confounding influences (i.e., additional masses, located at different positions); Cases 4 to 9 present damage-related frequency shifts, induced by stiffness reduction with no nonlinear sources; Cases 10 to 14 are affected by damage-like nonlinearities, which mimic a breathing crack model (with all the caveats explained before). Finally, Cases 15 to 17 are affected by nonlinearities and confounding influences.

## 4. Results

In this section, the extracted IMFs are extracted, visualised, and commented on; their comparability is also addressed. Then, their capabilities for ML-based SHM are tested. The parameters of the CEEMDAN, HVD, and VMD algorithms were set as follows:

1. For the CEEMDAN, the improved version of the code described in Ref. [30] has been utilised. This requires the following inputs:
  - The maximum number of sifting iterations allowed;
  - The number $N_{R}$ of WGN realisations;
  - The standard deviation σ of the applied noise.
  Here, a noise SD of σ=0.2 was applied for $N_{R}=15$, with a maximum of 3000 sifting iterations. Noteworthy, the original implementation of the code uses a pseudorandom number generator to generate the WGN realisations; this may affect the replicability of the results.
2. For the HVD, the original scripts released by Feldman for use with his book (Ref. [41]) have been applied. The user-defined parameters, which should be inserted manually, are:
  - The filter cut-off frequency (suggested to be in the range $0.05>f_{p}>0.005$);
  - The number *K* of modes to be extracted.
  In this study, $f_{p}$ was set to the recommended minimum value of 0.005. Regarding *K*, this parameter was set to 3 to represent the a priori knowledge of the user (i.e., that the mechanical system can be well-approximated by a three DoF oscillator).
3. For the VMD, the original code has been used (released in 2013 by the authors and downloadable at https://math.montana.edu/dzosso/code/index.html, last visited 20 October 2020). An official MatLab implementation has been made available in the Wavelet Toolbox (https://it.mathworks.com/help/wavelet/ref/vmd.html; last visited 20 October 2020) since early 2020; however, this latter version has not been tested here. The arbitrarily defined parameters, which may influence the final results, are
  - The initial values of $\left\{x_{k}^{1}\right\},\left\{\omega_{k}^{1}\right\},$ and $\lambda^{1}$: $\left\{x_{k}^{1}\right\}$ and $\lambda^{1}$, which are generally all assumed as zeros at the first iteration, while the frequencies in $\left\{\omega_{k}^{1}\right\}$ can be initially zeroed, uniformly distributed, or random;
  - α, the balancing parameter that defines the data-fidelity constraint;
  - τ, the time-step of the dual ascent that determines the updating of the Lagrangian multipliers (generally defined to have an exact reconstruction or to achieve denoising);
  - ϵ, the tolerance of the stopping criterion (generally around $10^{-6}$);
  - The number *K* of modes to be extracted.

The algorithm can be also used to remove the DC offset of the input signal if needed. In this study, the following conditions were considered: α=2000 (moderate bandwidth constrain), τ=0 (no strict fidelity enforcement), no DC offset correction, a uniform initial distribution of the $\left\{\omega_{k}^{1}\right\}$, and $\epsilon =10^{-7}$. The number *K* was set to 3 as explained before for the HVD for comparability.

Only for descriptive purposes, the cases portrayed here in the figures of this section refer to the response to the 25th realisation unless otherwise stated. All the 50 driving forces had similar amplitude and Power Spectral Density and led to comparable results. For conciseness, only the IMFs extracted from output channel #4 (i.e., at the highest floor) and for the first case (undamaged, linear baseline) are described in detail.

In the respective subsections, these are compared to the results from the fourteenth scenario (i.e., the most nonlinear case, with no added mass or reduced stiffness) to visualise how the algorithms compare with and without nonlinear distortions and to the other (linear and nonlinear) cases to address the effects of the structural changes. To take into account the input frequency range (20–150 Hz), all the algorithms were tested on the unfiltered, low-pass filtered, high-pass filtered, and band-pass filtered signals. All figures refer to the original (unfiltered) signals if not otherwise indicated. For interpretation of the references to colour in the figure legends, the reader is referred to the online version of this article.

### 4.1. Preliminary Results for Noise Sensitivity and Sensor Placement

Before being compared on the experimental dataset, the selected techniques have been tested on a numerical dataset for their robustness to artificially added measurement noise and their sensitivity to sensor placement.

For simplicity, the HVD and VMD algorithms were set to return only the first IMF; in both cases, this mode was basically corresponding to the first natural frequency. For comparability, the mode most similar mode (frequency-wise) was selected from the ones returned by the CEEMDAN algorithm. In almost all simulations, this was the 4th CEEMDAN IMF. The frequency corresponding to the peak amplitude of the identified IMF (hereinafter, the peak frequency $f_{p}$) was used as the feature of interest. This feature was expected to remain unvaried for unchanging structural conditions. Therefore, an arbitrary threshold was set to $\pm 10\%f_{p}$ and considered as the maximum error allowed.

**Figure 6:**
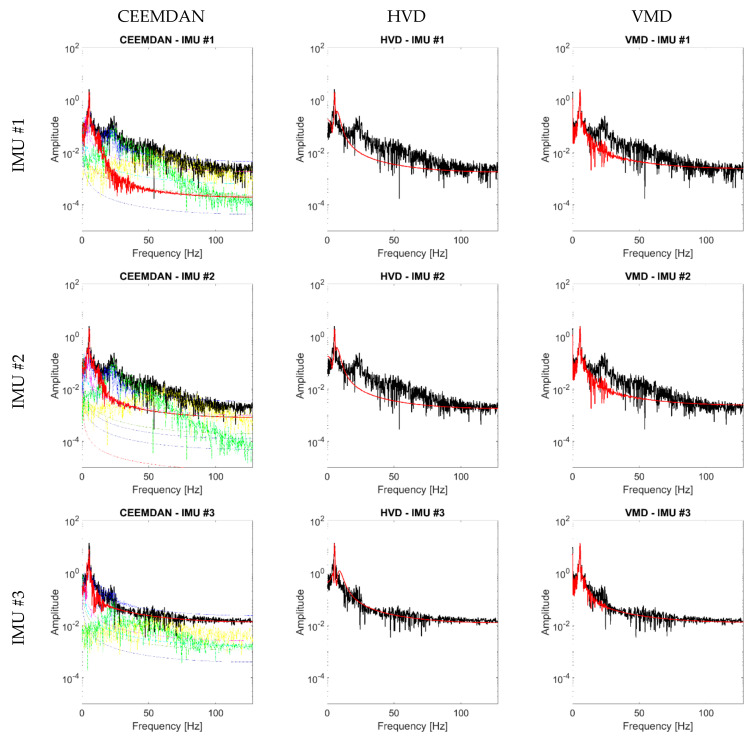

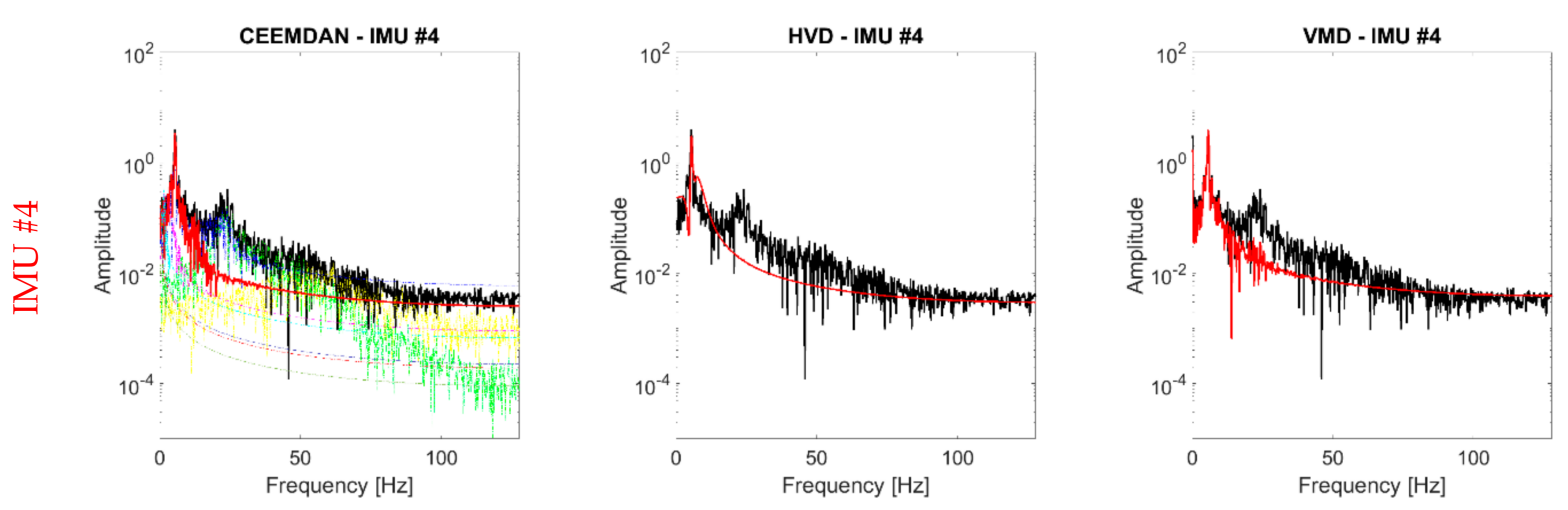
Selected IMF (1st IMF for HVD and Variational Mode Decomposition (VMD); 4th for CEEMDAN), in red, superimposed to the noise-free response signals (in black), according to the output channel considered. Only for the CEEMDAN algorithm, the other modes are indicated as coloured dash-dotted lines.

Figure 6 compares the selected IMF for CEEMDAN, HVD, and VMD for the four output channels depicted in Figure 4 and the noise-free scenario. One can observe that for this specific case, the identification of the selected mode was consistently centred around the fundamental frequency of the HAR wing spar, with negligible differences due to the specific sensor location.

**Figure 7:**
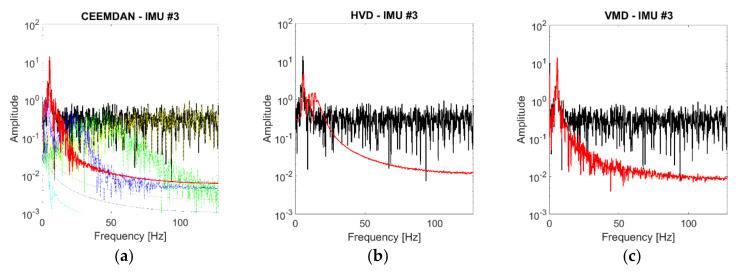
Selected IMF, superimposed in red to the response signals (in black), for the 3rd output channel. (**a**) CEEMDAN (4th IMF), (**b**) HVD (1st IMF), (**c**) VMD (1st IMF). The signals were corrupted with the maximum noise level (0.01 σ). Only for the CEEMDAN algorithm, the other modes are indicated as coloured dash-dotted lines.

**Figure 8:**
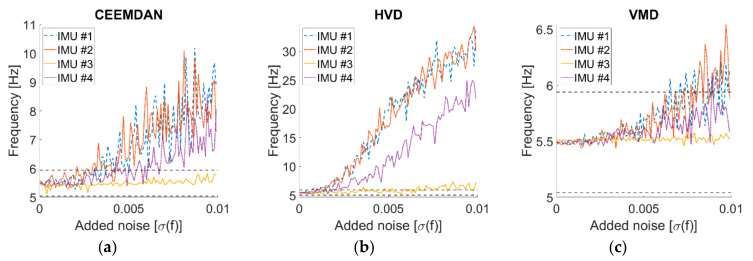
Effects of measurement noise on the identified peak frequencies. (**a**) Complete Ensemble Empirical Mode Decomposition with Adaptive Noise (CEEMDAN); (**b**) HVD; (**c**) VMD. The dashed horizontal lines indicate the upper and lower maximum error allowed.

As the noise level increases, the signal becomes more and more similar to a pure WGN (see Figure 7). This makes the identification procedure much more compelling. Figure 8 reports the peak frequency for increasing noise level (added according to what is specified in Section 3.1). In this case, the differences between the three techniques and the four channels are evident. As expected for the first flexural mode of a cantilevered beam-like structure, the recordings closer to the free tip (IMU #3) returned the identifications most robust to the additive noise in all cases.

For the CEEMDAN algorithm, the identifications from IMU #3 always fell in the range considered as acceptable, while the other sensors started to exceed the maximum threshold between 0.002 σ and 0.006 σ, depending on their distance from the clamped end.

For the HVD method, the values of the other IMUs started to increase from 0.001σ and rapidly escalated.

The VMD method returned the most robust identifications. Not only IMU #4 but also IMU #3 were stable or almost stable for all noise levels. The remaining two channels only started to diverge significantly after 0.065σ, and the absolute error was never larger than 1 Hz.

### 4.2. Computational Time Required

The computational times elapsed for running the non-optimised scripts on MatLab R2018b are discussed here, considering the experimental dataset.

**Figure 9:**
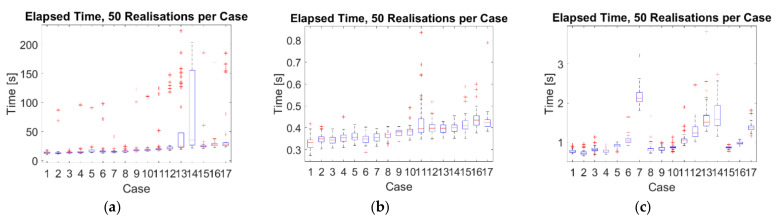
Boxplots of the computational time required by the algorithms. (**a**) CEEMDAN; (**b**) HVD; (**c**) VMD. On each box plot, the upper and lower whiskers extend to the most extreme values not accounted as outliers (these latter ones are indicated by the red ‘+’ symbol). The central mark indicates the median value, as indicated by the red horizontal line, and the bottom and top edges of the box represent the 25th and 75th percentiles, in this order.

All the runs were performed on an Intel^®^ Core™ i7-7700HQ CPU with a 2.80 GHz base frequency. The data pre- and post-processing phases have been excluded, focusing exclusively on the decomposition algorithms. The results, which refer to channel #4 and are averaged over 50 realisations per case, are compared in Figure 9. 

The candidate algorithms compared quite differently. The HVD, being by far the simplest algorithm, run almost 2.3 times faster than VMD and circa 40 times faster than CEEMDAN for the linear baseline. The CEEMDAN algorithm showed the largest variability due to its step involving the addition of random WGN. As expected, for all three options, the cases with the added nonlinearity were the ones most computationally demanding. The VMD encountered major difficulties in dealing with case #7 as well.

Even if this study focusses on offline data interpretation, these results are also important in consideration of potential applications for real-time damage detection and online modal identification, which require minimal computational expenses [61].

### 4.3. CEEMDAN IMFs

The CEEMDAN is the only procedure that automatically set the number of its IMFs. The exact number was found to vary depending on the structural conditions. Considering all realisations, channels, and cases, the range spans from a minimum of 11 IMFs (extracted <1% of the times) to a maximum of 15 (3.41%). The decomposition returned 12 or 13 IMFs with an overall occurrence respectively of 41.12% and 48.74%. Results with >15 IMFs (2.35% of the total) can be safely considered as outliers; these happened very often in cases #14 (and to a lesser extent in #13, #15, #16, and #17), due to the noise-like nonlinear distortions being modelled as signal components, reaching 50 IMFs or more. This demonstrates that even with all the improvements with respect to the standard EMD, the CEEMDAN algorithm still suffers an over-decomposition issue. These non-physical IMFs severely hampers the capabilities of the CEEMDAN algorithm.

**Figure 10:**
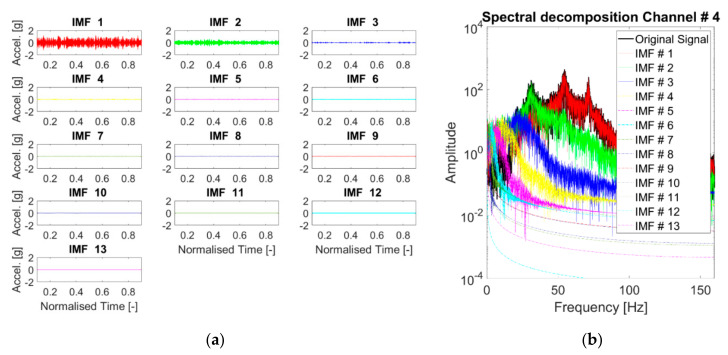
CEEMDAN-Extracted IMFs. (**a**) Time domain; (**b**) acceleration Power Spectral Densities (PSDs). Response to the 25th White Gaussian Noise (WGN) input, channel #4, undamaged baseline (case #1). The red, green, and blue solid lines correspond to the 1st, 2nd, and 3rd IMF, in this order; the other IMFs are indicated by the dash-dotted lines.

In all cases, only a few of the first IMFs are of practical interest, as can be seen from Figure 10, which reports the resulting modes in the time and frequency domains for the linear baseline. The first IMF englobes the 2nd and the 3rd eigenmodes of the structure, while the second IMF mostly captures the 1st mode. Thus, the algorithm failed to separate the LNMs of the system.

Any IMF after the third one has a much lower amplitude, and its energy content is bounded to the lower frequencies. These components can even be removed for denoising purposes. Therefore, only K=1,2, and 3 will be discussed in more detail hereinafter.

Regarding the instantaneous frequency, the IFs of the first two IMFs were found to be almost stable as expected, while the other extracted modes are subject to fluctuations that do not have physical meaning.

Regarding the effects of filtering, removing the frequencies out of the input range (f<20 Hz and >150 Hz) was not particularly beneficial nor detrimental for the CEEMDAN algorithm. The number of the extracted IMFs was not sensibly reduced. The first modes were limitedly affected, while residual IMFs changed noticeably in both the frequency and the time domain; however, these latter components are of almost null utility for SHM purposes whatsoever.

**Figure 11:**
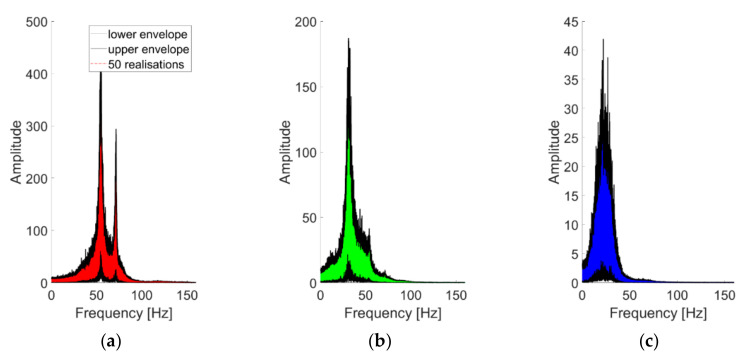
(**a**–**c**) The first three IMFs (in this order) as extracted from 50 realisations and superimposed. Channel #4, undamaged baseline (case #1).

To investigate the statistical stability of the procedure, the first three IMFs as extracted from 50 realisations of the baseline scenario are superimposed in Figure 11. The three components show a good consistency for the several repetitions; the same considerations can be extended to all the linear (damaged or undamaged cases). The nonlinear case showed a much higher variability, especially for IMF #1.

### 4.4. HVD IMFs

**Figure 12:**
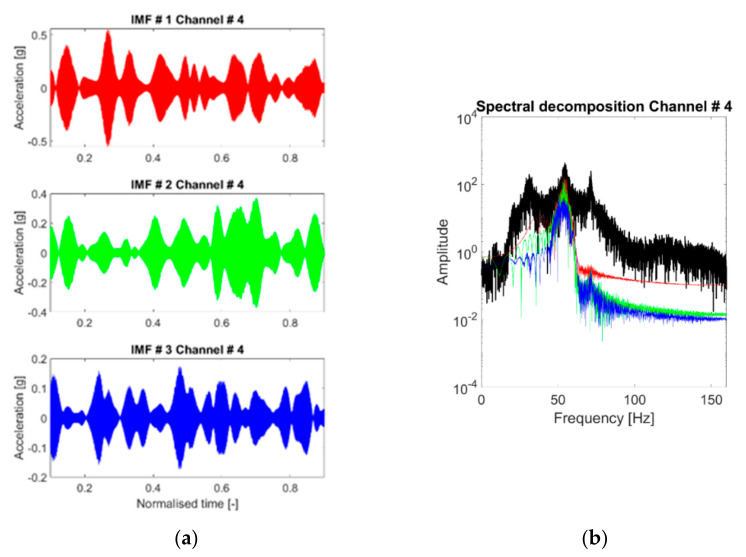
Acceleration PSDs of the extracted IMFs. Response to the 25th WGN input, channel #4, undamaged baseline (case #1). HVD-Extracted IMFs. (**a**) Time domain; (**b**) acceleration PSDs. Response to the 25th WGN input, channel #4, undamaged baseline (case #1).

**Figure 13:**
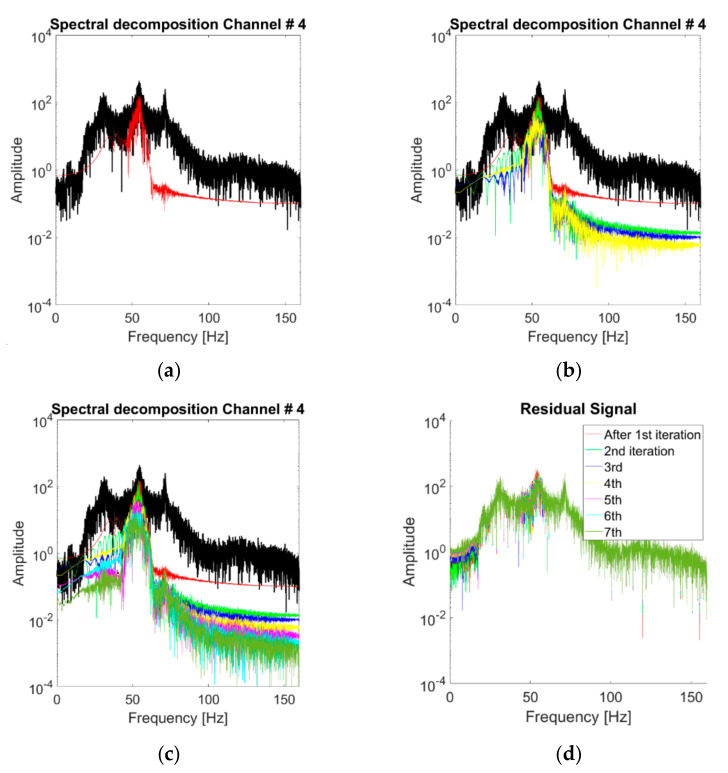
Extracted modes for varying K (response to the 25th WGN input, channel #4, undamaged baseline). From (**a**–**d**) *K* = 1, 4 and 7. (**d**) Residual signals for seven iterations.

For K=3, Figure 12b represents the spectral decomposition of the response at the channels considered for case #1 (linear baseline). These modes are portrayed one by one in the time domain in Figure 12a (notice the different scales on the y-axis). The HVD-extracted IMFs are not similar to the structure’s eigenmodes, as foreseeable, since this decomposition is energy-related rather than frequency-related. All the extracted IMFs have basically the same frequency support, surrounding the main frequency peak (i.e., the 2nd natural frequency of the system). This is most probably because even after the subtraction of the main component, the frequency corresponding to this vibrational mode remains the most prominent energy-wise. This point reverberates in the effects of varying *K*, as represented in Figure 13. The first IMF to be extracted coincides with the 2nd natural frequency of the structure. Further increasing the value of *K* does not add useful information about the other two vibrational modes. The reason is the one highlighted above and represented in Figure 13d. After seven iterations, the energy distribution of the residual signal is still qualitatively similar to the original one frequency-wise; hence, any new IMF is extracted around the same $\omega_{k}$ at ≈54 Hz. 

The impact of the nonlinear distortions in HVD-extracted IMFs is less evident at first sight than for CEEMDAN-extracted IMFs. Generally, the IMFs’ energy distribution in the frequency domain is comparable with the ones of the linear cases. This can be explained since a major part of the IMFs’ energy is bandlimited in a portion of the spectrum less directly affected by the nonlinearity (this point will be further addressed in the next subsections). Therefore, this finding is specific to the dataset considered here and cannot be generalised.

The IF time histories are quite unstable, with fluctuations of more than ±10 Hz. Moreover, due to the known limitations of the HT, the identifications near the signal ends are unreliable.

For what concerns the filtering, at least for the case study here investigated, this did not generate any noticeable improvement or counterproductive effect, with the resulting IMFs having inconsequential differences in the frequency domain (even if slightly more pronounced and potentially non-negligible in the time domain).

**Figure 14:**
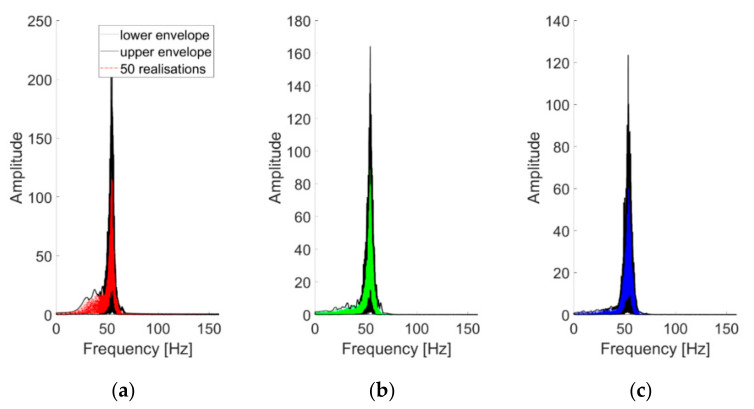
(**a**–**c**) The first three IMFs (in this order) as extracted from 50 realisations and superimposed. Channel #4, undamaged baseline (case #1).

To conclude, the intra-scenario variability is addressed in Figure 14. The IMFs are consistent for the 50 slightly different inputs when applied to the same structural configuration, with the variability slightly larger at the lower frequencies. The same findings apply to the damaged and nonlinear cases as well.

### 4.5. VMD IMFs

**Figure 15:**
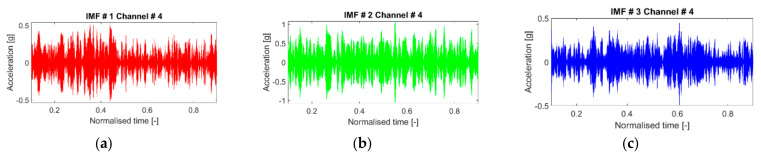
(**a**–**c**) Extracted IMFs in the time domain. Response to the 25th WGN input, channel #4, undamaged baseline (case #1).

**Figure 16:**
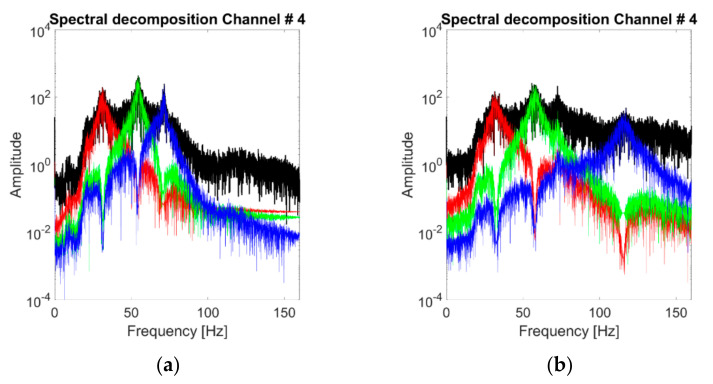
Acceleration PSDs of the extracted IMFs. Response to the 25th WGN input, channel #4. (**a**) Undamaged baseline (case 1); (**b**) case #14 (most nonlinear scenario).

The discussion about the VMD band-limited IMFs follows the same steps seen in the subsections before. The IMFs extracted at the fourth channel for the baseline scenario are individually portrayed in Figure 15. Figure 16a shows the spectral decomposition of the corresponding PSD. In comparison with the HVD and CEEMDAN, the extracted IMFs are very similar to the LNMs of the structure. However, there are some exceptions: for instance, in the nonlinear case #14, for channel output #4, the algorithm fails to properly isolate the third vibrational mode (Figure 16b), capturing instead a superharmonic peak at circa 115 Hz. The third eigenmode was eventually isolated by increasing K to 4.

**Figure 17:**
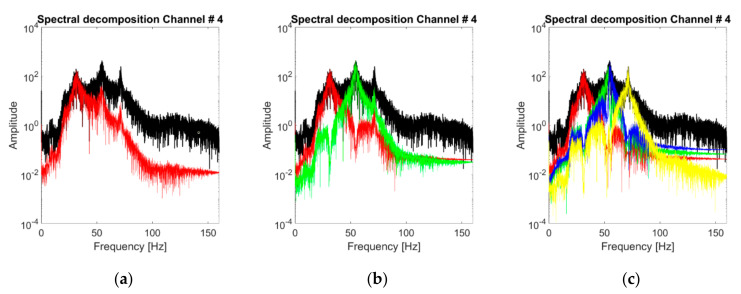
Extracted modes for varying *K* (response to the 25th WGN input, channel #4, undamaged baseline). From (**a**–**c**) K=1, 2 and 4.

The investigation on varying the parameter *K* is shown in Figure 17. The VMD algorithm reacts well in the sense that it captures a good approximation of the first eigenmode for K=1 and a good estimation of the other two eigenmodes for K=2 and K=3. On the other hand, increasing K≥3 produces no physically meaningful modes in the linear case of Figure 17c, while it was found to successfully isolate super- and sub-harmonics in other nonlinear scenarios, as explained before.

The IF time histories of the IMFs were found to be generally very stable and not noticeably affected by the presence of large nonlinear distortions—even the superharmonics, when isolated by increasing K≥4 in cases #10 to #17, are properly tracked instant by instant. The corresponding centre frequencies also showed good stability for the several recordings.

**Figure 18:**
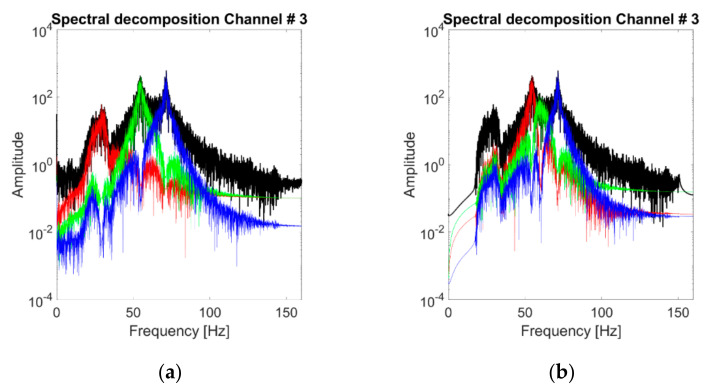
Effects of filtering on the VMD procedure (response to the 25th WGN input, channel #3). Case #1 (**a**) unfiltered; (**b**) band-pass filtered (20–150 Hz).

Unexpectedly, it was observed that filtering out the frequencies below 20 Hz caused the algorithm to not correctly isolate the first natural frequency on some (not frequent) occasions, as reported in Figure 18 for the 3rd channel (baseline case). In this specific case, the issue was solved by reducing the high-pass cut-off frequency to 12 Hz or less. This point suggests that frequency peaks too close to the cut-off frequencies may be more difficult to isolate, which is most probably due to the presence of attenuated content in the nearby frequencies. The same occurrence, while not frequently encountered, was noticed for some nonlinear and damaged scenarios as well. Therefore, the VMD returned overall better results when applied to the signal without filtering it.

**Figure 19:**
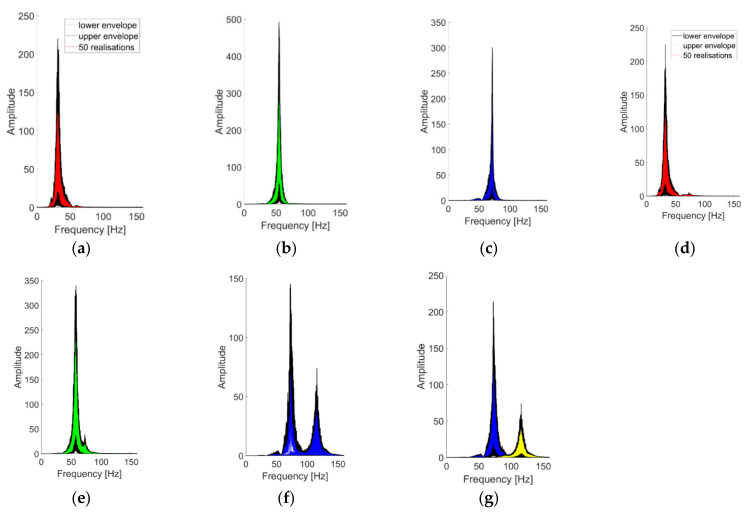
The first three IMFs (in this order) as extracted at channel #4 from 50 realisations and superimposed. (**a**–**c**) undamaged baseline (case #1); (**d**–**f**) case #14; (**g**) 3rd and 4th IMFs for case #14 (considering K=4; 1st and 2nd IMFs almost unchanged).

Concluding this first discussion about the extracted IMFs, the statistical stability of the VMD results is portrayed in Figure 19 by superposing the 50 realisations for each IMF in the frequency domain. In the linear cases, the identification procedure returns stable and consistent modes, with limited variability amplitude-wise and very limited variability frequency-wise. For the nonlinear scenarios, the mislabeling between the 3rd natural mode and the 1st superharmonic caused a superposition of two distinguished IMFs (Figure 19f). The issue was easily solved by increasing K to 4. This almost completely solved any ambiguity (Figure 19g).

### 4.6. Comparability of the Extracted Modes

**Figure 20:**
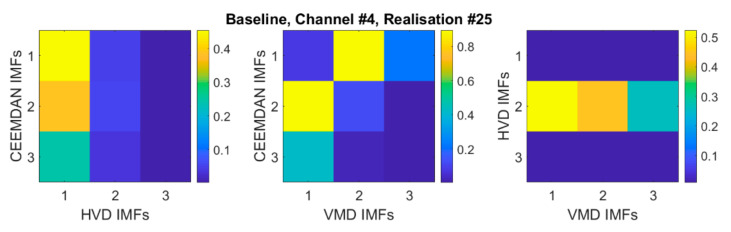
Correlation coefficients for the comparison of the extracted IMFs (only the first three IMFs are considered for the CEEMDAN algorithm).

In Figure 20, the IMFs extracted by means of the CEEMDAN, HVD, and VMD algorithms are compared to establish if and how much similar they are. This was done by considering the correlation coefficient of each signal pair. The reported results refer to the 25th realisation of the undamaged baseline. From the first matrix, it is visible that for the CEEMDAN and HVD methods, the first IMF is the most similar, even if it is still quite different in absolute terms. The second matrix shows instead how the 1st CEEEMDAN IMF compares very well with the 2nd VMD IMF and vice versa. This is coherent with what has been observed before, as the 1st CEEMDAN IMF englobed both the 2nd and the 3rd eigenmodes, while the 2nd CEEMDAN IMF corresponded roughly to the 1st normal mode. Finally, from the last matrix (on the right), one can notice the second LNM, which is recurrent in all HVD modes and captured by the 2nd IMF of the VMD approach. Similar outcomes were encountered for the other scenarios as well.

### 4.7. Sensitivity of the Extracted IMFs to Damage

Having established the similarities and differences among the IMFs extracted with the three different techniques, it is now necessary to address their reliability as DSFs for Machine Learning. All the proposed techniques allow for precise time-frequency analysis, which is useful for both real-time continuous monitoring and the a posteriori analyses of short-timed extreme conditions such as earthquakes, explosions, typhoons etc. on the structure.

**Figure 21:**
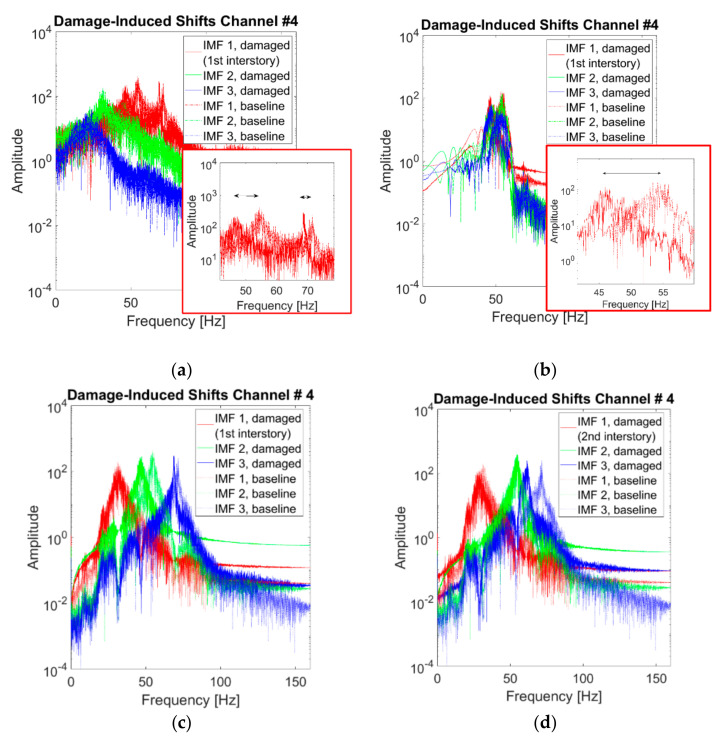
Comparison of extracted IMFs with and without damage. (**a**) CEEMDAN first three IMFs, case #1 vs. case #5, with a zoom on the first IMF; (**b**) HVD BLIMFs, case #1 vs. case #5, with a zoom on the first IMF; (**c**) VMD BLIMFs, case #1 vs. case #5; (**d**) VMD BLIMFs, case #1 vs. case #7. In all subplots, the dash-dotted curves refer to the undamaged baseline (case #1) and the solid lines refer to the damage conditions. Red, green, and blue correspond to the first, second, and third IMFs, in this order. Channel #4, 25th realisation.

**Figure 22:**
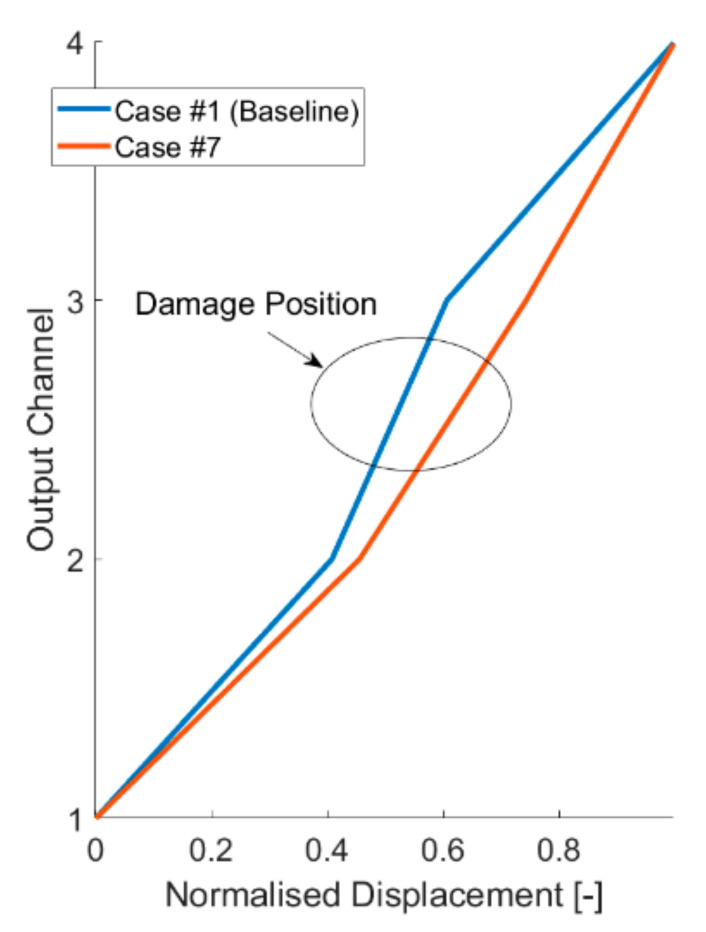
Deflection shapes extracted from the corresponding first VMD band limited IMF (centred and normalised to the maximum displacement), baseline, and case #7. The damage location is highlighted in the ellipse.

Damage can be detected in several modes. The frequency-based approaches rely on changes in the PSD distribution. Figure 21 highlights how this is easily doable with CEEMDAN, HVD, and VMD-extracted IMFs. Figure 21a shows the CEEMDAN IMFs, where the major frequency shifts (linked to the 2nd and 3rd natural frequencies) are absorbed into the first IMF for all the damaged scenarios. In Figure 21b, all the HVD BLIMFs are very sensitive to the changes of case #5 and insensitive to the ones of case #7, as they are all centred around the 2nd eigenfrequency. This proves that different IMFs extracted with different techniques react more or less markedly to specific typologies and locations of the damage. For instance, for case #5 (linear response, strongly damaged at the 1st inter-storey), the frequency shift of the 2nd VMD BLIMF is clear (Figure 21c), while this is less evident for the 3rd and the 1st BLIMFs. In case #7 (Figure 21d), the damage-induced effects are prominent for the 3rd BLIMF and almost unnoticeable in the other two. This also reflects the different positions of the stiffness reduction. For these reasons, the extracted IMFs can be even used for more specific damage assessment tasks, such as damage localisation, exploiting their differences and peculiarities as generally done for modal parameters and especially mode shapes [48]. In this sense, mode shape-based approaches rely on the vibrational modes of the structure, which can be in turn obtained from the modal components of the signal. Considering the VMD-extracted BLIMFs as a sufficiently good approximation of the investigated system’s eigencomponents, the structural deflection shapes can be qualitatively estimated from them. Figure 22 shows an example of damage localisation, exploiting the deflection shapes corresponding to the first identified VMD BLIMF and the known location of the four output channels. However, proper damage localisation using these extracted features will need further investigation before being established beyond any reasonable doubts.

**Figure 23:**
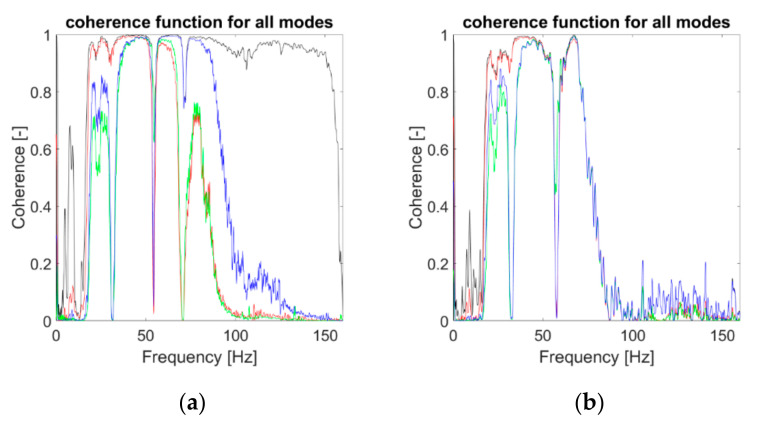
Results from the nonlinearity test. (**a**) Case #1 (undamaged baseline) (**b**) case #14. Black line: coherence of the whole recording (channel #4, 25th realisation). Red, green, and blue lines: 1st, 2nd, and 3rd VMD-extracted IMFs (in this order).

Finally, assuming the damage as a pointwise source of nonlinearity in an otherwise linear system, any nonlinear test can be run over the single IMFs as normally done for the whole signal. The magnitude-squared coherence function [62], defined between the input force and the output channel #4, is reported in Figure 23 for the three VMD-extracted BLIMFs compared to the whole signal. It is evident how, for case #14 (i.e. the one with the strongest nonlinearity), the 3rd BLIMF is much more affected than the 2nd IMF, while the first component is almost unaltered. This reflects what was seen previously, since a large portion of the frequency support of the 3rd BLIMF falls in the distorted region (roughly from 70 Hz upward). This aspect will be further recalled in the following subsection as well.

Therefore, depending on the intended use, several IMFs, especially the ones obtained through VMD, can be exploited to discern (or even locate) operational changes, the presence of nonlinearities, or stiffness reductions.

### 4.8. Uses for ML-Based SHM

This last part aims to illustrate how the DSFs extracted from the signal components can be used to train an ML algorithm to perform automatically continuous monitoring. Indeed, algorithms such as the EMD are appreciated for pattern recognition problems [63], which are the basis for ML-based SHM.

**Figure 24:**
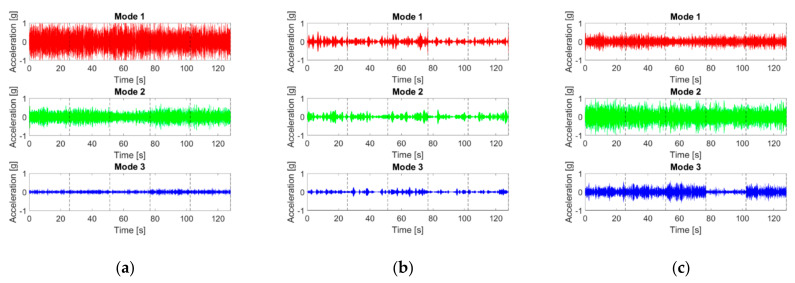
(**a**–**c**) CEEMDAN, HVD, and VMD IMFs (in this order) as extracted from the concatenated signal recorded at channel #4 for the 25th realisation.

To replicate the settings of Ref. [59], the recorded accelerations from Cases #1, #3, #7, #14, and #17 have been concatenated. This is intended to simulate time-varying structural changes such as operational conditions (with mass overloads) and damage inception (both linear and nonlinear). The resulting modes are portrayed in Figure 24. As before, only the response at the fourth (top storey) channel for the 25th WGN input is reported for brevity. It can be noticed that the amplitude of the HVD IMFs is much lower than the one of their VMD and CEEMDAN counterparts.

The IF time histories of the IMFs extracted from the undamaged baseline have been used to define a “normality” condition for the target structure. To ensure the replicability of the results, a simple yet effective approach, the Gaussian Process Regression (GPR; [64]), has been utilised. This approach is well-known in the SHM community for surrogate modelling and has been proved valid by decades of extensive use [65,66,67]. All 50 realisations have been used to define the normality model and the corresponding 3 σ thresholds. Only for the HVD method, to avoid the HT edge effects, 5% of the data at both ends have been removed from the training dataset (this step was unnecessary for both CEEMDAN and VMD).

**Figure 25:**
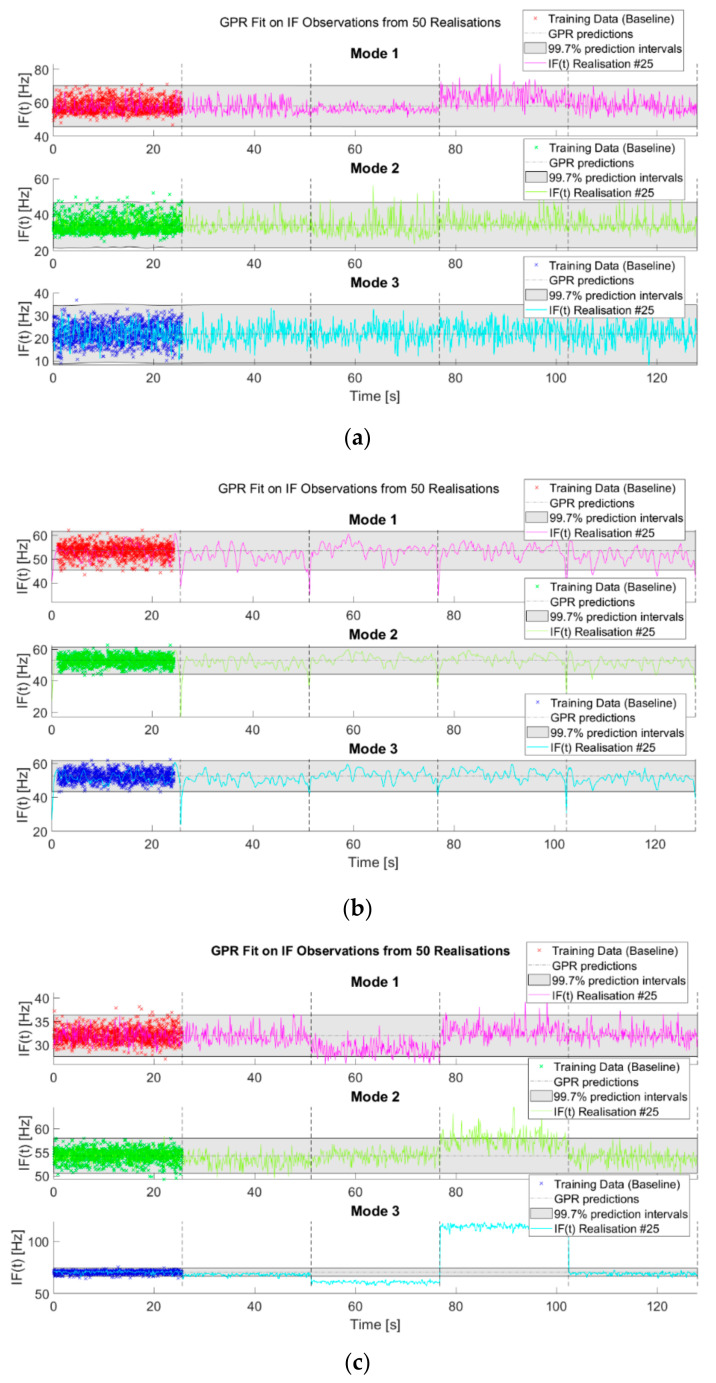
Results from the outlier detection. Normal models defined from the instantaneous frequencies (IFs) of the extracted modes over 50 realisations. (**a**) CEEMDAN; (**b**) HVD; (**c**) VMD.

Figure 25 shows the results of this analysis. As explained before, the intent of this paper is not to address any specific ML technique for anomaly detection; the aim is to compare the results obtained from the use of mode-related features. In this simple case presented here, the CEEMDAN-extracted IMFs (Figure 25a) are the only ones with noticeable (even if very small) fluctuations of the IF in their baseline model. The first IMF seems to be relatively sensitive to damage and structural changes, as it slightly deviates from the predicted value (dash-dotted grey line) for case #7 and more markedly for the nonlinear case #14. However, it falls mostly at any instant in the 99.7% confidence interval (grey area). The 2nd and 3rd IMFs seem to be unchanged by the added mass or the inserted pointwise source of nonlinearity. This can be both an advantage (e.g., for masonry structures, where many material and geometrical nonlinearities cause noise-like, damage-unrelated distortions) or a limitation (e.g., being insensitive to breathing cracks’ effects). For the 2nd IMF, it is noticeable, as decreasing the stiffness causes the DSF to trespass the threshold value for a few instants in case #7. Nevertheless, such deviation from the normality model is arguably too small and time limited to signify damage with good confidence.

The HVD-extracted modes (Figure 25b) did not show good potential for damage detection, remaining unaltered for varying structural conditions and quite unstable overall. VMD-extracted modes (Figure 25c) showed more encouraging results. The IF of the first IMF differs appreciably from the expected value for case #7 (linear damage), even if it is still in the 3σ range almost everywhere. On the other hand, it is quite unaffected by the presence of the nonlinear distortions and by the added masses. Conversely, mode #2 is more affected by the added mass (case #3) than by the reduced stiffness (case #7), even if the deviation from the predicted values is well under the alarm threshold. On the other hand, it is quite sensitive to the most nonlinear scenario of case #14. Finally, the third IMF is the most sensitive one to both linear and nonlinear damage, trespassing the confidence interval for case #7 and even more markedly for case #14, while remaining under the threshold for the cases with the additional masses. This finding confirms what was reported in Ref. [59], where the bumper’s impacts were found to affect the most the third eigenmode of the structure.

## 5. Discussion and Conclusions

In this work, data-adaptive mode decomposition techniques have been investigated for SHM purposes. Departing from the broad set of proposals available in the scientific literature, the selection was narrowed down to the three methods—the EMD, HVD, and VMD algorithms. These candidates have been considered to be representative of the whole current state of the art, due to their popularity and the several similarities shared with other strictly related approaches.

The three algorithms have been methodically investigated and compared. Their theoretical and practical strong and weak points have been thoroughly discussed. All the parameters and settings have been reported for the reproducibility of the results.

The examined IF time histories were found to be suitable for ML-based damage detection, even in the presence of noise-like distortions induced by structural nonlinearities and when concatenated to mimic sudden structural changes and signal nonstationarity. The key findings of this comparative study can be enlisted as follows:

1. Regarding the choice between the data-driven or manual selection of the number K of modes to be extracted, for structural engineering purposes, the second option should be preferred. The number of relevant vibrational modes of a target structure can be easily estimated or guessed; on the other hand, having a fixed number of modes greatly simplifies the comparison between different measurements. This also makes the detection of (most likely damage-related) anomalies easier and more reliable.
2. When comparing the IMFs extracted from different recordings, it is necessary to check their comparability to avoid blunders.
3. Filtering the acceleration THs of the structure under random excitations did not improve significantly any of the tested algorithms, or at least not for the specific case investigated here.
4. The CEEMDAN algorithm can be used to isolate the most important characteristics of the target signal, yet it is greatly affected by measurement noise and nonlinear distortions.
5. The HVD is better suited for time-domain applications rather than frequency-domain ones. It has a remarkable role for nonlinear SDoF systems where it can efficiently isolate distinct (linear or nonlinear) terms for forced or free vibrations. It is also well suited for the analysis of nonstationary responses to external impulses and the extraction of energy-related damage sensitive features. However, it is less apt for systems with multiple degrees of freedom and/or to track damage-related frequency shifts.
6. The VMD approach performed overall better than the CEEMDAN and HVD techniques for tracking frequency shifts.

To conclude, all the reviewed approaches have their strong points that can serve specific tasks. For vibration-based SHM purposes, the authors of this comparative analysis will finally suggest VMD as a viable option with several noteworthy advantages and few practical and theoretical limitations. Hopefully, this comparative analysis on a well-documented experimental case study will provide some suggestions for the practitioners in search of the most fitting approach for their applications, as well as some hints for researchers committed to developing new alternatives. 

The authors are committed to performing similar comparative analyses for other promising (even if slightly less known) algorithms and also for wavelet-based approaches. Real-time applications will be of great interest as well.

## Figures and Tables

**Table 1:** Main features of the described methods.

Feature	EMD	HVD	VMD
Basis	Self-Adaptation	Self-Adaptation	Prior Determination
Frequency	Difference, Local	Difference, Global	Difference, Global
Characterisation domain	Energy-Time	Energy-Time-Frequency	Energy-Time-Frequency
Apt for nonlinear systems?	Yes	Yes	Yes
Apt for nonstationary signals?	Yes	Yes	No
Feature extraction	Yes	Yes	Yes
Theoretical foundations	Empirical	Complete Theory	Complete Theory

**Table 2:** Geometrical and mechanical properties of the wing spar Finite Element Model (FEM).

Property	Value	Measurement Unit
Free length (clamp to tip) $l_{TIP}$	706.00	mm
Thickness t	2.00	mm
Max width at the Clamped section $b_{\mathrm{MAX}}$	180.00	mm
Min width at the tip section $b_{\mathrm{MIN}}$	17.04	mm
Young’s modulus	$59.02\times 10^{3}$	MPa
Density	2893.07	$\mathrm{kg}/m^{3}$
Damping ratio	0.86	%
Poisson’s ratio	0.26	-

**Table 3:** The experimental dataset.

Case	Description
1	Undamaged baseline
2	Undamaged with confounding influences; added mass of 1.2 kg at the base
3	Undamaged with confounding influences; added mass of 1.2 kg at the 1st floor
4	Linear damage; 87.5% stiffness reduction in one column of the 1st inter-storey
5	Linear damage; 87.5% stiffness reduction in two columns of the 1st inter-storey
6	Linear damage; 87.5% stiffness reduction in one column of the 2nd inter-storey
7	Linear damage; 87.5% stiffness reduction in two columns of the 2nd inter-storey
8	Linear damage; 87.5% stiffness reduction in one column of the 3rd inter-storey
9	Linear damage; 87.5% stiffness reduction in two columns of the 3rd inter-storey
10	Nonlinear damage; distance between bumper and column tip 0.20 mm
11	Nonlinear damage; distance between bumper and column tip 0.15 mm
12	Nonlinear damage; distance between bumper and column tip 0.13 mm
13	Nonlinear damage; distance between bumper and column tip 0.10 mm
14	Nonlinear damage; distance between bumper and column tip 0.05 mm
15	Nonlinear damage with confounding influences; Bumper 0.20 mm from column tip, 1.2 kg added at the base
16	Nonlinear damage with confounding influences; Bumper 0.20 mm from column tip, 1.2 kg added on the 1st floor
17	Nonlinear damage with confounding influences; Damaged bumper 0.10 mm from column tip, 1.2 kg added on the 1st floor

## Data Availability

The data from the numerically simulated HAR wing used to support the findings of this study are available from the corresponding author upon request.

## Appendix A

The following Table A1, Table A2 and Table A3 report all the advantages and disadvantages of the three methods (EMD, HVD, and VMD, in the same order) as expressed by the authors of the papers reviewed here in this study.

**Table A1:** Summary of the advantages and disadvantages of EMD and derived algorithms.

EMD, EEMD, or CEEMDAN	Advantages	Relatively simple to apply and implement. Time-tested approach (applied extensively for more than 20 years). Fully data-driven: it does not require any prior knowledge of the structure under examination [68]. It automatically defines the optimal number of modes depending on the properties of the signal. On the other hand, different signals may return a different number of modes, making direct comparison difficult or unreliable [68]. It can operate with any data length [10] (except for computational issues). Thanks to its adaptivity, it has been demonstrated as an efficient tool for signal denoising in the case of both homoscedastic or heteroscedastic noise [69]. The damage effects can be limited and/or better appreciated in a subset of modes or even in a single IMF (which can be automatically selected accordingly to a feature of interest; see e.g., Ref. [70]). Several different stopping criteria exists in the scientific literature for the sifting process (e.g., a Standard Deviation threshold [7], the S-number [71], the Threshold Method [72], or the Energy Difference Tracking [73] criterion). It can be seen as a dyadic filter bank, similarly to the Discrete Wavelet Decomposition [74]. The extracted modes are hierarchically ordered: the first IMF contains the highest-frequency component, while, at any step, information of lower-frequency components is left into the residual signal [10]. This latter point can be also exploited to estimate the Hurst exponent H [74] of the investigated time series.
	Disadvantages	The EMD suffers from mode-mixing; this issue is only partially solved by the EEMD and CEEMDAN implementations. The CEEMDAN algorithm remains very sensitive to additive noise [47]. The modes may have a wide frequency band (thus not being a monocomponent term as expected). This is especially true for the first IMF [75]. Real-life signals are sampled, i.e., the continuous-time signal is reduced to a discrete-time reduction of the same during the measurement. A maximum/minimum of the original continuous signal may fall between two sampled points and therefore not be observed or correctly identified. This can undermine the correct identification of the extrema and therefore of their upper and lower envelopes [68]. The quality of the upper and lower envelopes depends on the choice of (1) The locality rule for peak finding; (2) The interpolation function. Different variants may lead to different estimations of the IMF [68] (e.g., the effects of spline interpolation are addressed in detail in Ref. [76]). For datasets of length n, the order of time complexity of the classic EMD is $O\left(n\;log\;n\right)$; i.e., it is equivalent to the traditional Fourier transform (but with a larger factor) [77]. This makes it less computationally efficient than other techniques such as ITD [78] and not applicable as it is for real-time, online monitoring [79]. The IMF components of the signal may have poor orthogonality; this limits their usefulness as a basis of the dynamic system [80]. The global, recursive sifting procedure does not allow for backward error correction [12]. It struggles to separate wide-band modes with overlapping spectra [81] and close modes [82] (generally, at least one octave of distance in the frequency domain is needed [47]). Some low-energy components may be inseparable [54,83]. Close modes can be difficult to isolate as well [54,83]. Some fictitious (computational) low-amplitude components may appear at low frequencies [82]. These have been reported to “spoil” [83] the time-frequency map, making the interpretation by a human user more difficult. Some parameters, such as the maximum number of sifting iterations allowed, the number of WGN realisations, and the standard deviation of the applied noise, are still selected manually and may affect the results. The pseudo-randomly generated WGN realisations strongly affect the results, the computational time, and effort required. This can cause instability issue at the software level as well. The procedure suffers from the end effects, overshoots, or undershoots typical of the HT [54].

**Table A2:** Summary of the advantages and disadvantages of HVD.

HVD	Advantages	Based on a single and simple signal processing technique (the Hilbert transform), with a clear analytical formulation and backed by solid physical principles. On the other hand, the HT is affected by all the issues explained in Section 2.1.1. Fully data-driven. It returns an envelope-based hierarchy of modes (the first mode has the highest varying amplitude, the following ones are ordered for decreasing amplitude), rather than frequency-based [54] (depending on the intended use, this can be both an advantage or a downside). The resulting IMFs are analytically defined with specific characteristics. It shows better frequency resolution in comparison to EMD, with greater capabilities to decompose closer frequencies (this aspect is deeply investigated in Ref. [47]). Nevertheless, it is still unable to detect very close modes (i.e., if the difference between the two natural frequencies is less than $f_{p}$ [10]). In at least one study [84], it has been proved to be more computationally efficient than an EMD-based option for detrending and baseline wander removal. Due to its significantly lower computational cost, the HVD has been deemed as “more appealing” [85] than EMD for real-time applications, such as for online monitoring. The number *K* of modes to be extracted is selected arbitrarily and can be varied as desired. On the other hand, there is no guarantee that the user’s assumption corresponds to the real number of independent components. In this regard, Feldman [10] suggests using a limit value of the standard deviation difference, computed from the two consecutive iteration results, as a stopping criterion for the sifting process. The data-driven automatic setting of K is still considered to be beyond the current capabilities [47].
	Disadvantages	It has been proved to be as sensitive to additive noise as the EMD algorithm [47]. Being physically based, the method needs some theoretical requirements [10]: (1) All $A_{k}\left(t\right)$ and $\omega_{k}\left(t\right)$ must be slowly varying; (2) Any $A_{k}\left(t\right)$ amplitude must be significantly larger than the following $A_{k+1}\left(t\right)$ (3) The duration of $x\left(t\right)$ must include at least some (possibly, several) periods of the slowest component. That said, the approach suits well most of the signal of interest for SHM, including nonstationary vibrations and highly nonlinear mechanical systems with large harmonic distortions (as long as they are almost periodic). For these reasons, the algorithm is not applicable for analysing intermittent or non-oscillating signals [54]. Due to the above prescriptions, the approach cannot extract aperiodic components of the signal [10]. Requires relatively long recordings, while the EMD can operate with shorter acquisitions [10,86]. Depending on the aim, an amplitude-based hierarchy may be preferable to a frequency-based one. However, since the shift in the natural frequencies is one of the main damage-sensitive features, the other way around is generally better suited for SHM. Less accurate than EMD for the abrupt envelope and/or frequency instantaneous variations such as frequency jumps and crossings [47,86]. The cut-off frequency $f_{p}$, while being the only required parameter, is critical for the results and cannot be easily defined with absolute certainty. For technical reasons, the sampling frequency $f_{s}$ is strongly suggested to be in the range $\left(20-80\right)\times f_{0}$, with $f_{0}$ being the fundamental frequency of the signal [41].

**Table A3:** Summary of the advantages and disadvantages of VMD.

VMD	Advantages	Has a solid theoretical background and analytical formulation. Fully data-driven. The method is non-recursive, and the decomposed modes are extracted concurrently rather than iteratively (differently from EMD and HVD) [12,50]. This allows for error balancing between them through a joint-optimisation scheme. Does not require specific improvements to deal with measurement noise. The similarities of the mode updating procedure with the Wiener filter suggests that the approach has some optimality in dealing with measurement noise [12]. The VMD has been proved to be not only robust to noise but also sensitive in detecting weak side-band DSFs that are often masked by the background noise [83]. The extracted sub-signals have their own centre frequency and, for a sufficiently long time interval, can be considered as pure harmonics [12]. The resulting IMFs are bandwidth-limited, differently from the ones obtained from EMD. This narrow-band IMFs allows a proper single-mode decomposition [12], similarly to the HVD method [10]. The VMD has been proved to be well-suited for signal denoising and detrended fluctuation analysis, with an order of time complexity comparable to that of EMD [87]. It has been recently proposed in implementations for real-time pattern recognition, proving a major efficiency in comparison with EMD and wavelet-based approaches [88]. Does not require to predefine the filter bank boundaries [12]. The VMD has been shown to outperform EMD for intra-wave signal analysis [83]. The equivalent filter bank of the VMD has a Wavelet Packet (WP)-like structure, whereas the EMD has a more Wavelet-like equivalent filter bank. The former one results in more subdivisions in the time-frequency space, allowing (at least theoretically) to extract more features with higher resolution [51], allowing also to distinguish close modes [80]. As for the HVD, the number *K* of modes to be extracted is defined manually, which allows some flexibility but at the cost of adding more arbitrariness to the process.
	Disadvantages	As for the HVD, the assumptions on the definition of the modes do not allow extracting non-periodic components. Differently from EMD, HVD, and other approaches such as the Discrete Wavelet Transform, it does not automatically return a hierarchy of modes (neither amplitude nor frequency ordered), even if this can be achieved easily in post-processing. Not appliable to non-$C^{1}$ discontinuous signals (e.g., sawtooth signals) [12]. Arguably, the instantaneous frequency of such signals is not physically very meaningful; yet it can still be useful for the analysis of signals from systems with abrupt structural changes (potentially even damage-induced). Struggles to decompose wide-band modes with overlapping spectra [80]. The method requires some user-defined parameters (α, τ, ϵ), which may affect the results. These are more than e.g., for HVD, which only requires to define the cut-off frequency.
